# Supplementary material for: A Normalization Protocol Reduces Edge Effect in High-Throughput Analyses of Hydroxyurea Hypersensitivity in Fission Yeast
Source: Biomedicines. 2023 Oct 18;11(10):2829. doi: 10.3390/biomedicines11102829 (PMC10604075; doi:10.3390/biomedicines11102829)
Supplement: Supplementary file 1 [file biomedicines-11-02829-s001.zip › biomedicines-2639056-supplementary.pdf]

biomedicines

MDPI

Supplementary Materials

Table S1. MER strains used in high-throughput ROTOR screening in this paper. The table lists out doxorubicin-resistance strains used in high-throughput ROTOR screening with HU and the relative coordinates of the positions where cells were pinned at 96- or 384-spot setting.

MER Strain No.	Relative coordinates at 96-spot setting	Relative coordinates at 384-spot setting	Genetic Background
1	A2	A3, A4, B3, B4	*Δyox1*
2	A3	A5, A6, B5, B6	*Δssb3*
3	A4	A7, A8, B7, B8	*ΔSPAC6G9.14*
4	A5	A9, A10, B9, B10	*Δsce3*
5	A6	A11, A12, B11, B12	*ΔSPAC11E3.12*
6	A7	A13, A14, B13, B14	*Δdph2*
7	A8	A15, A16, B15, B16	*Δcsn1*
8	A9	A17, A18, B17, B18	*Δvps901*
9	A10	A19, A20, B19, B20	*Δdad5*
10	A11	A21, A22, B21, B22	*Δdad2*
11	A12	A23, A24, B23, B24	*ΔSPBC19G7.10c*
12	B1	A25, A26, B25, B26	*Δppx1*
13	B2	A27, A28, B27, B28	*Δvph2*
14	B3	A29, A30, B29, B30	*ΔSPBC16H5.13*
15	B4	A31, A32, B31, B32	*Δmug166*
16	B5	A33, A34, B33, B34	*ΔSPCC1840.09*
17	B6	C1, C2, D1, D2	*Δgit5*
18	B7	C3, C4, D3, D4	*Δhap2*
19	B8	C5, C6, D5, D6	*Δvps35*
20	B9	C7, C8, D7, D8	*Δada1*
21	B10	C9, C10, D9, D10	*Δrhp55*
22	B11	C11, C12, D11, D12	*Δrsc4*
23	B12	C13, C14, D13, D14	*Δest1*
24	C1	C15, C16, D15, D16	*Δrrd1*
25	C2	C17, C18, D17, D18	*ΔSPCC18.02*
26	C3	C19, C20, D19, D20	*Δase1*
27	C4	C21, C22, D21, D22	*Δarp42*
28	C5	C23, C24, D23, D24	*Δyaf9*

MER Strain No.	Relative coordinates at 96-spot setting	Relative coordinates at 384-spot setting	Genetic Background
29	C6	C25, C26, D25, D26	*Δrhp54*
30	C7	C27, C28, D27, D28	*Δnpp106*
31	C8	C29, C30, D29, D30	*ΔSPAC823.10c*
32	C9	C31, C32, D31, D32	*Δnht1*
33	C10	C33, C34, D33, D34	*Δapl6*
34	C11	E1, E2, F1, F2	*Δngg1*
35	C12	E3, E4, F3, F4	*Δgcn5*
36	D1	E5, E6, F5, F6	*Δclr5*
37	D2	E7, E8, F7, F8	*Δdad3*
38	D3	E9, E10, F9, F10	*Δcor1*
39	D4	E11, E12, F11, F12	*Δcsn2*
40	D5	E13, E14, F13, F14	*Δnrm1*
41	D6	E15, E16, F15, F16	*Δduo1*
42	D7	E17, E18, F17, F18	*Δmhf1*
43	D8	E19, E20, F19, F20	*Δspc19*
44	D9	E21, E22, F21, F22	*Δtim11*
45	D10	E23, E24, F23, F24	*Δcbp6*
46	D11	E25, E26, F25, F26	*Δiec1*
47	D12	E27, E28, F27, F28	*Δpmd1*
48	E1	E29, E30, F29, F30	*Δabo1*
49	E2	E31, E32, F31, F32	*Δada2*
50	E3	E33, E34, F33, F34	*Δccr4*
51	E4	G1, G2, H1, H2	*Δrsc1*
52	E5	G3, G4, H3, H4	WT
53	E6	G5, G6, H5, H6	*Δlcf1*
54	E7	G7, G8, H7, H8	*Δppr1*
55	E8	G9, G10, H9, H10	*Δtup12*
56	E9	G11, G12, H11, H12	*Δcph2*
57	E10	G13, G14, H13, H14	*Δcoq6*
58	E11	G15, G16, H15, H16	*Δcoq7*
59	E12	G17, G18, H17, H18	*Δmms1*
60	F1	G19, G20, H19, H20	*Δgit1*
61	F2	G21, G22, H21, H22	*Δphp3*
62	F3	G23, G24, H23, H24	*Δerd2*

**MER Strain No.**	**Relative coordinates at 96-spot setting**	**Relative coordinates at 384-spot setting**	**Genetic Background**
63	F4	G25, G26, H25, H26	*Δcoq2*
64	F5	G27, G28, H27, H28	*Δcoq4*
65	F6	G29, G30, H29, H30	*Δrpa12*
66	F7	G31, G32, H31, H32	*Δdps1*
67	F8	G33, G34, H33, H34	*Δcox19*
68	F9	I1, I2, J1, J2	*Δmcl1*
69	F10	I3, I4, J3, J4	*Δarp5*
70	F11	I5, I6, J5, J6	*Δcdt2*
71	F12	I7, I8, J7, J8	*Δrad32*
72	G1	I9, I10, J9, J10	*Δrhp51*
73	G2	I11, I12, J11, J12	*Δdad1*
74	G3	I13, I14, J13, J14	*Δmhf2*
75	G4	I15, I16, J15, J16	*Δrad24*
76	G5	I17, I18, J17, J18	*Δctp1*
77	G6	I19, I20, J19, J20	*Δapl5*
78	G7	I21, I22, J21, J22	*Δcox6*
79	G8	I23, I24, J23, J24	*Δcay1*
80	G9	I25, I26, J25, J26	*Δmfm2*
81	G10	I27, I28, J27, J28	*ΔSPAC17H9.08*
82	G11	I29, I30, J29, J30	*Δtom7*
83	G12	I31, I32, J31, J32	*Δatd1*
84	H1	I33, I34, J33, J34	*Δcoq10*
85	H2	K1, K2, L1, L2	*Δcoq3*
86	H3	K3, K4, L3, L4	*Δcaf1*
87	H4	K5, K6, L5, L6	*Δarp8*
88	H5	K7, K8, L7, L8	*Δies6*
89	H6	K9, K10, L9, L10	*Δies4*
90	H7	K11, K12, L11, L12	*Δies2*
91	H8	K13, K14, L13, L14	*Δiec3*
92	H9	K15, K16, L15, L16	*Δrav1*
93	H10	K17, K18, L17, L18	WT
94	H11	K19, K20, L19, L20	*Δcds1*

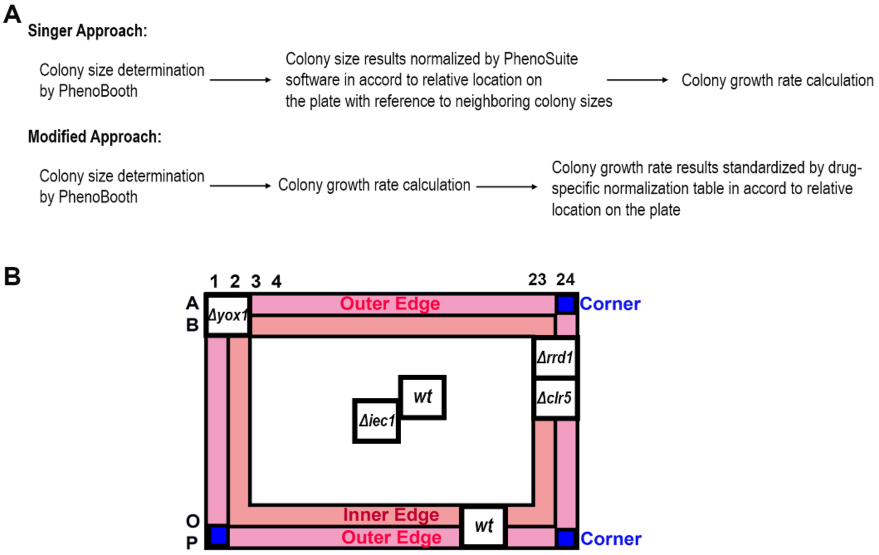

C DXR strain 1 (*Δyox1*); HU sensitive in spotting

		**Before PhenoSuite Normalization**			**After PhenoSuite Normalization**		
Coordinate at 384-spot setting	Presence of Edge Effect	Colony size	ROTOR HU sensitivity relative to *wt* pinned at center of the plate	ROTOR HU sensitivity relative to *wt* pinned at edge of the plate	Colony size	ROTOR HU sensitivity relative to *wt* pinned at center of the plate	ROTOR HU sensitivity relative to *wt* pinned at edge of the plate
A1	Yes	194	No	Yes	66	Yes	Yes
A2	Yes	153	No	Yes	89	Yes	Yes
B1	Yes	168	No	Yes	98	Yes	Yes
B2	No	107	Yes	Yes	107	Yes	Yes
	Average	155	No	Yes	90	Yes	Yes

D DXR strain 24 (*Δrrd1*); HU resistant in spotting

		**Before PhenoSuite Normalization**			**After PhenoSuite Normalization**		
Coordinate at 384-spot setting	Presence of Edge Effect	Colony size	ROTOR HU sensitivity relative to *wt* pinned at center of the plate	ROTOR HU sensitivity relative to *wt* pinned at edge of the plate	Colony size	ROTOR HU sensitivity relative to *wt* pinned at center of the plate	ROTOR HU sensitivity relative to *wt* pinned at edge of the plate
C23	No	107	Yes	Yes	107	Yes	Yes
C24	Yes	173	No	Yes	103	Yes	Yes
D23	Yes	115	Yes	Yes	115	Yes	Yes
D24	No	174	No	Yes	104	Yes	Yes
	Average	142	No	Yes	107	Yes	Yes

E DXR strain 36 (*Δclr5*); HU sensitive in spotting

		**Before PhenoSuite Normalization**			**After PhenoSuite Normalization**		
Coordinate at 384-spot setting	Presence of Edge Effect	Colony size	ROTOR HU sensitivity relative to *wt* pinned at center of the plate	ROTOR HU sensitivity relative to *wt* pinned at edge of the plate	Colony size	ROTOR HU sensitivity relative to *wt* pinned at center of the plate	ROTOR HU sensitivity relative to *wt* pinned at edge of the plate
E23	No	120	Yes	Yes	120	Yes	Yes
E24	Yes	205	No	Yes	122	No	Yes
F23	No	140	No	Yes	140	No	No
F24	Yes	120	Yes	Yes	122	No	Yes
	Average	146	No	Yes	126	No	Yes

F DXR strain 46 (*Δiec1*); HU resistant in spotting

		**Before PhenoSuite Normalization**			**After PhenoSuite Normalization**		
Coordinate at 384-spot setting	Presence of Edge Effect	Colony size	HU sensitivity relative to *wt* pinned at center of the plate	HU sensitivity relative to *wt* pinned at edge of the plate	Colony size	HU sensitivity relative to *wt* pinned at center of the plate	HU sensitivity relative to *wt* pinned at edge of the plate
G12	No	182	No	Yes	182	No	No
G13	No	204	No	Yes	204	No	No
H12	No	190	No	Yes	190	No	No
H13	No	197	No	Yes	197	No	No
	Average	193	No	Yes	90	No	No

G *wt*; Pinned at center of the plate

Coordinate at 384-spot setting	Presence of Edge Effect	Colony size before PhenoSuite	Normalized colony size after PhenoSuite
I9	No	113	113
I10	No	118	118
J9	No	127	127
J10	No	129	129
	Average	121	121

H *wt*; Pinned at edge of the plate

Coordinate at 384-spot setting	Presence of Edge Effect	Colony size before PhenoSuite	Normalized colony size after PhenoSuite
O17	No	161	161
O18	Yes	219	128
P17	Yes	264	154
P18	Yes	313	106
	Average	239	138

Figure S1. Built-in normalization function of Singer PhenoSuite® software over-correct colony size readings at 384-spot setting. (A) Flowchart to indicate normalization protocol of Singer PhenoSuite® software and modified protocol adapted by this paper (B) A map illustrating relative pinning position of multiple yeast strains of different deletion backgrounds at a control plate with no HU. (C-H) PhenoSuite® software equips with a program script to normalize potential edge effects with reference to colony values (at 96th hour of incubation on drug plate) of neighboring colonies on the agar plate. Nonetheless, PhenoSuite® script detects most colonies impacted by "edge effects" but over-corrects with those which is closer to the corners of rectangular plate (e.g., *Δyox1* colonies at A1 or A2 positions), giving a misleading average colony growth rate and strain sensitivity detection even with multiple replicates.

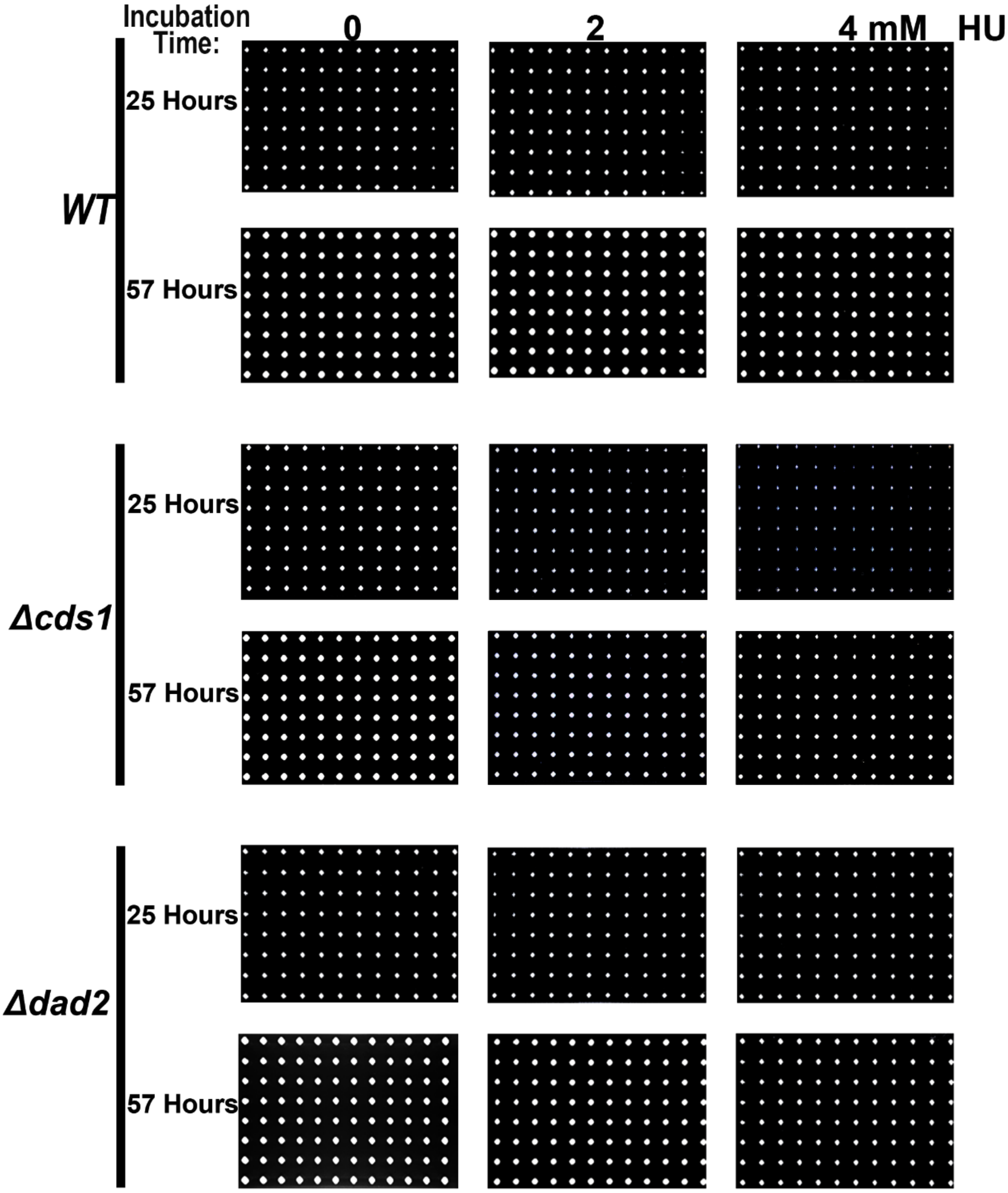

Figure S2. Edge effect was not visually observable at definite timepoint at 96-spot setting. WT, *Δcds1* and *Δdad2* cells were pinned onto agar plate at spot density of 96 with 0, 2 and 4mM HU. Edge effects were not visually observed with snapshots of growth patterns of cells after 25-hr (representing early growth phase) and 57-hr (representing late growth phase) incubation at 30°C.

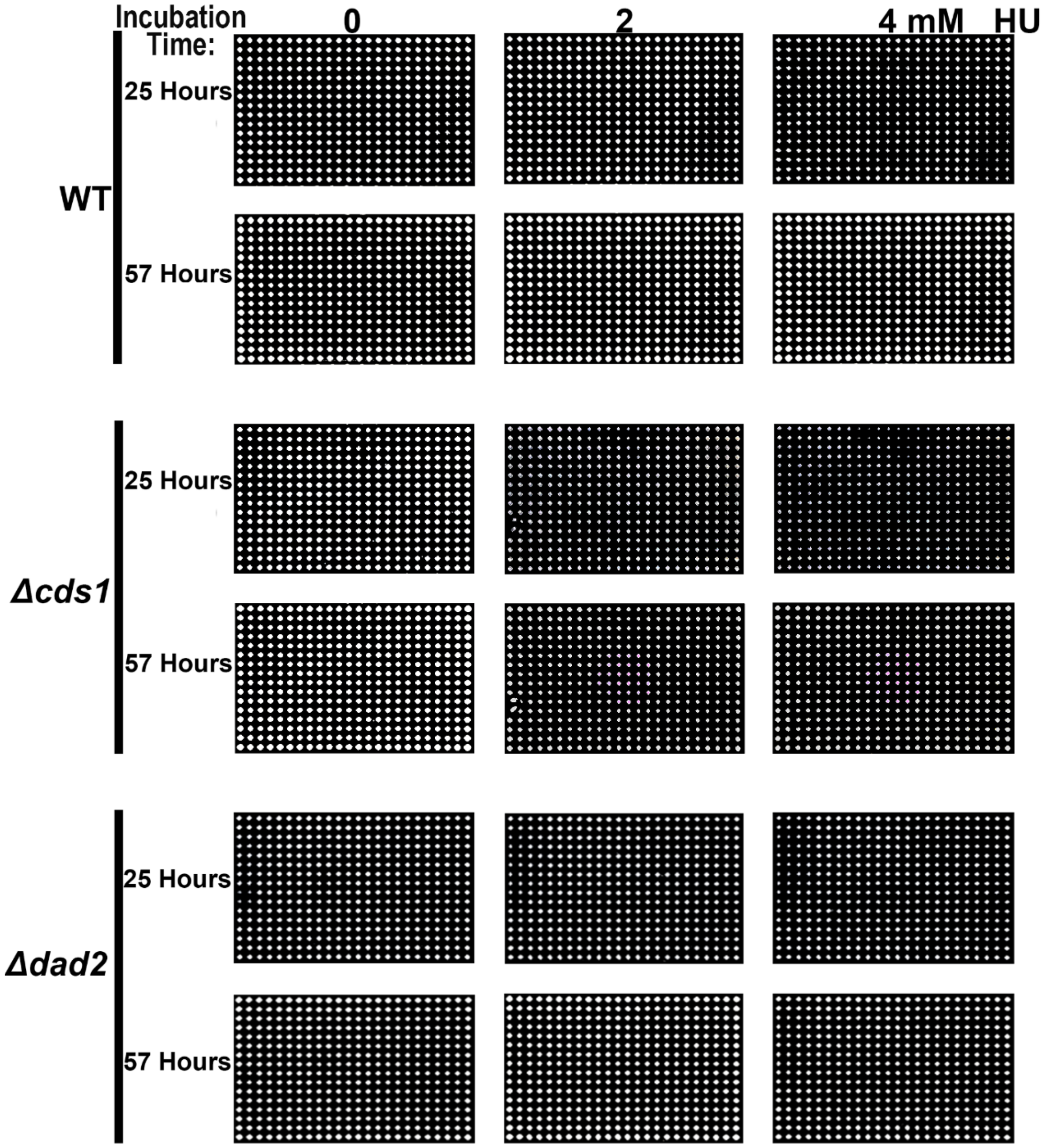

Figure S3. Edge effect was visually observable at definite timepoint at 384-spot setting. WT, *Δcds1* and *Δdad2* cells were pinned onto agar plate at spot density of 384 with 0, 2 and 4mM HU. Edge effects were not visually observed with snapshots of growth patterns of cells after 25-hr (representing early growth phase) and 57-hr (representing late growth phase) incubation at 30°C, without further data processing.

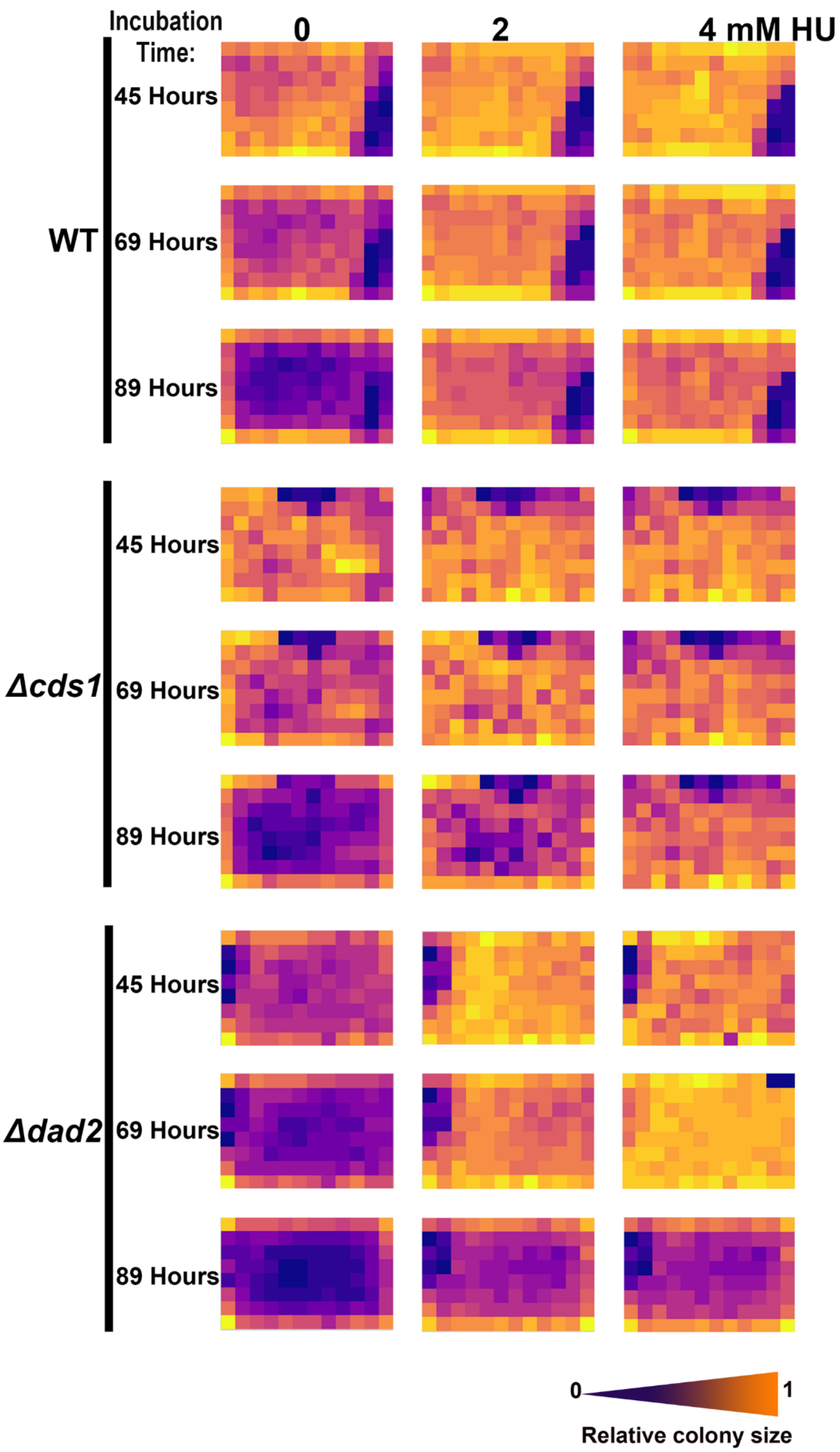

Figure S4. Edge effect was detected with a heatmap of colony size at late phase at 96-spot setting. Colony size values were measured by PhenoSuite® software to generate heatmaps illustrating relative colony size at different position of control and HU plates Edge effect (in term of colony size value) became observable only in late growth phase in both control and drug plates at 96-spot setting, which is consistent with growth rate value.

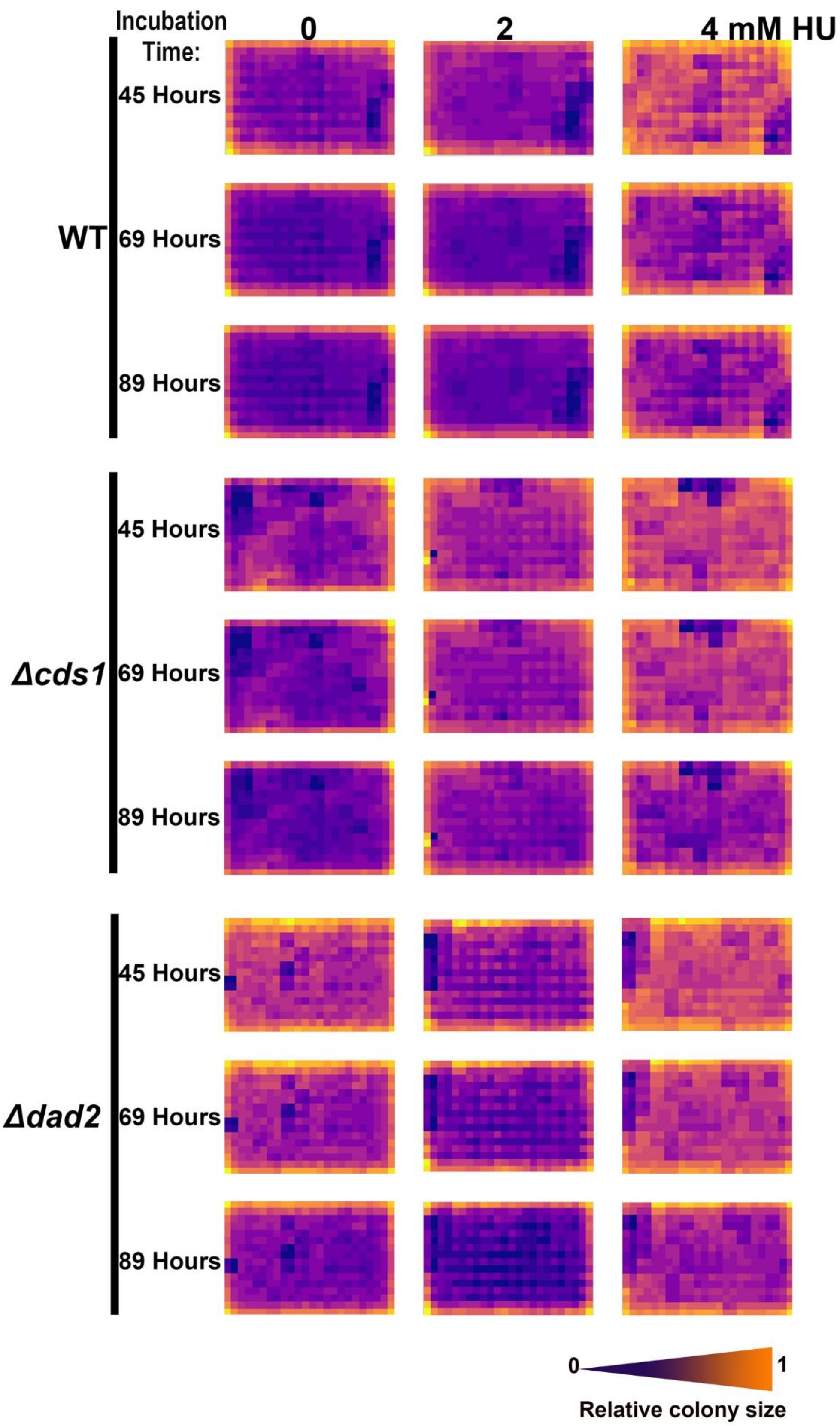

Figure S5. Edge effect was detected with heatmaps of colony size at late phase at 96-spot setting. Colony size values were measured by PhenoSuite® software to generate heatmaps illustrating relative colony size at different position of control and HU plates. Edge effect (in term of colony size value) is generally prominent in all plates at spot density of 384 from 45th hours after incubation.

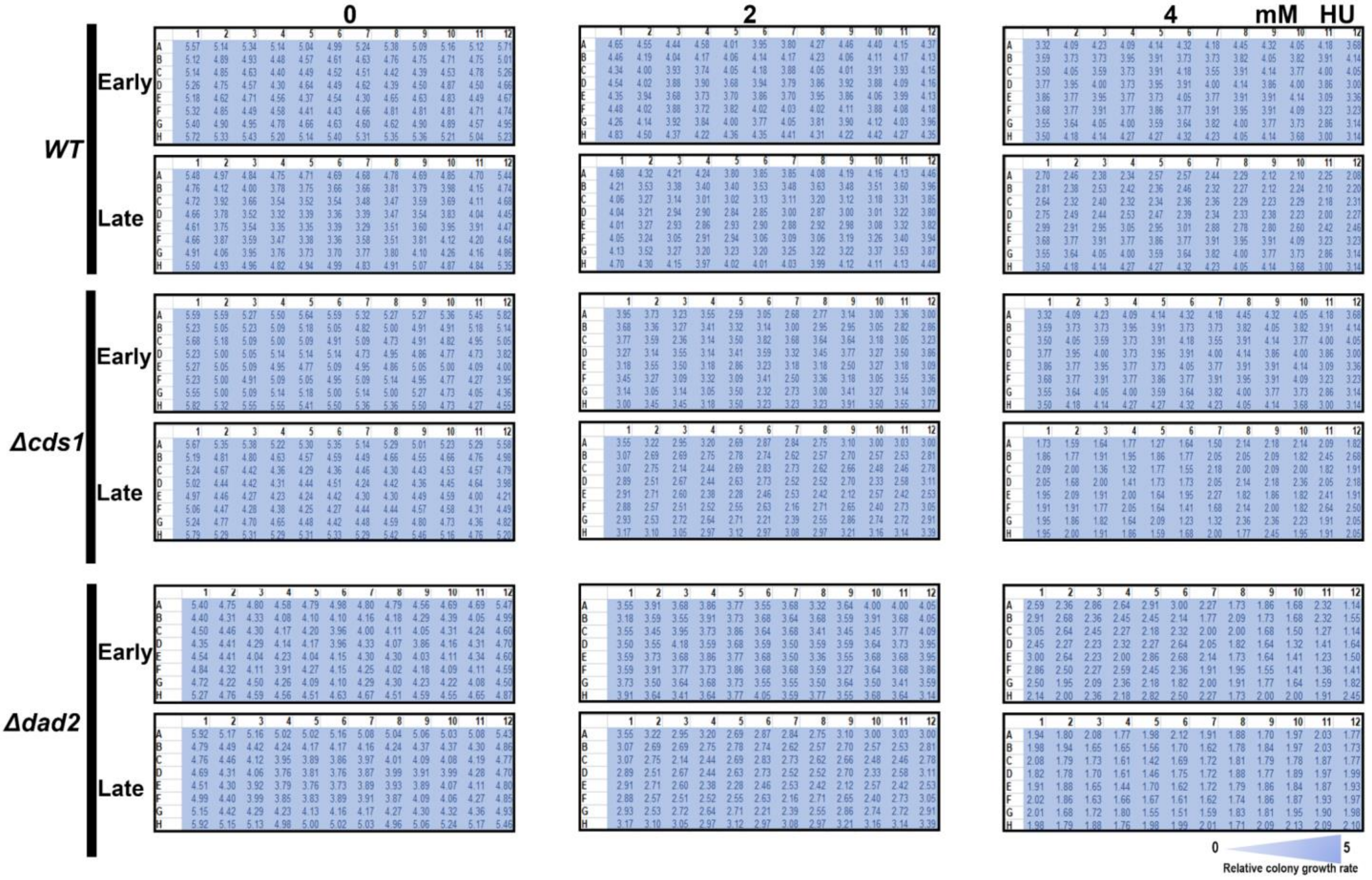

Figure S6. Limited edge effect was detected with a heatmap of growth rate at late phase at 96-spot setting. (A) WT, (B) *Δcds1* and (C) *Δdad2* cells were pinned onto agar plate at spot density of 96 with 0, 2 and 4mM HU. Early and late growth rate were calculated from the slope of growth curve generated from the PhenoSuite® and organized into heatmaps with annotation of relative growth rate at different loci. A minute level of edge effects was detected at late phase in terms of colony growth rate in 96-spot setting.

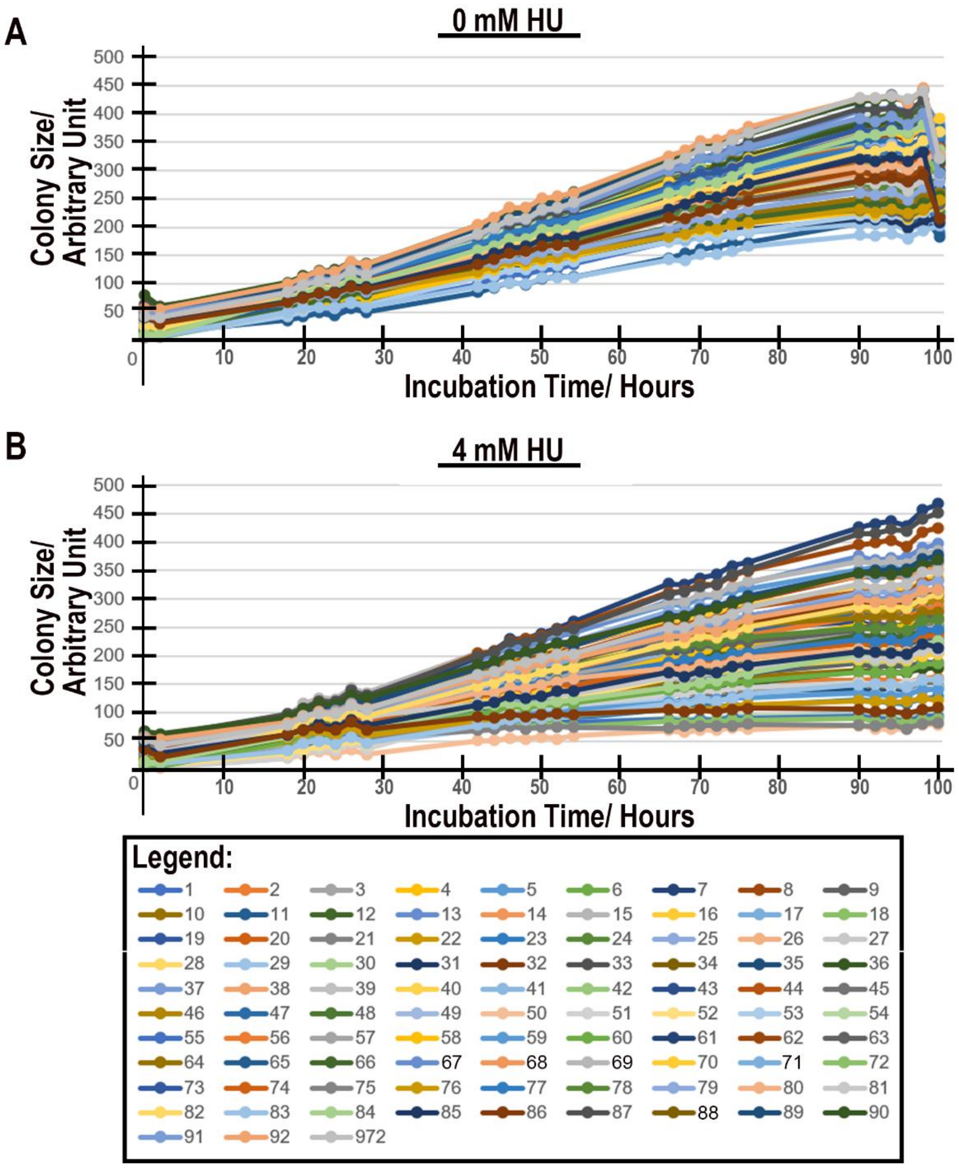

C

	ROTOR Early Growth Rate vs Day 3 Spotting	ROTOR Late Growth Rate vs Day 7 Spotting
False Positive %	5.3	1.8
False Negative %	8.8	8.8
Accuracy%:	93.4	95.6
Precision%:	94.7	98.2
Recall%:	94.7	94.9

Figure S7. ROTOR-based HU screening at 96-spot setting. (A) Example of growth curves of all MER strains pinned on control plate (without HU) at 96-spot setting. (B) Example of growth curves of all MER strains pinned on 4mM HU plate at 96-spot setting. (C) A high level of false rate was observed for ROTOR-based HU screening at low pinning density, which demonstrated a need for an optimization protocol for improving screening accuracy.

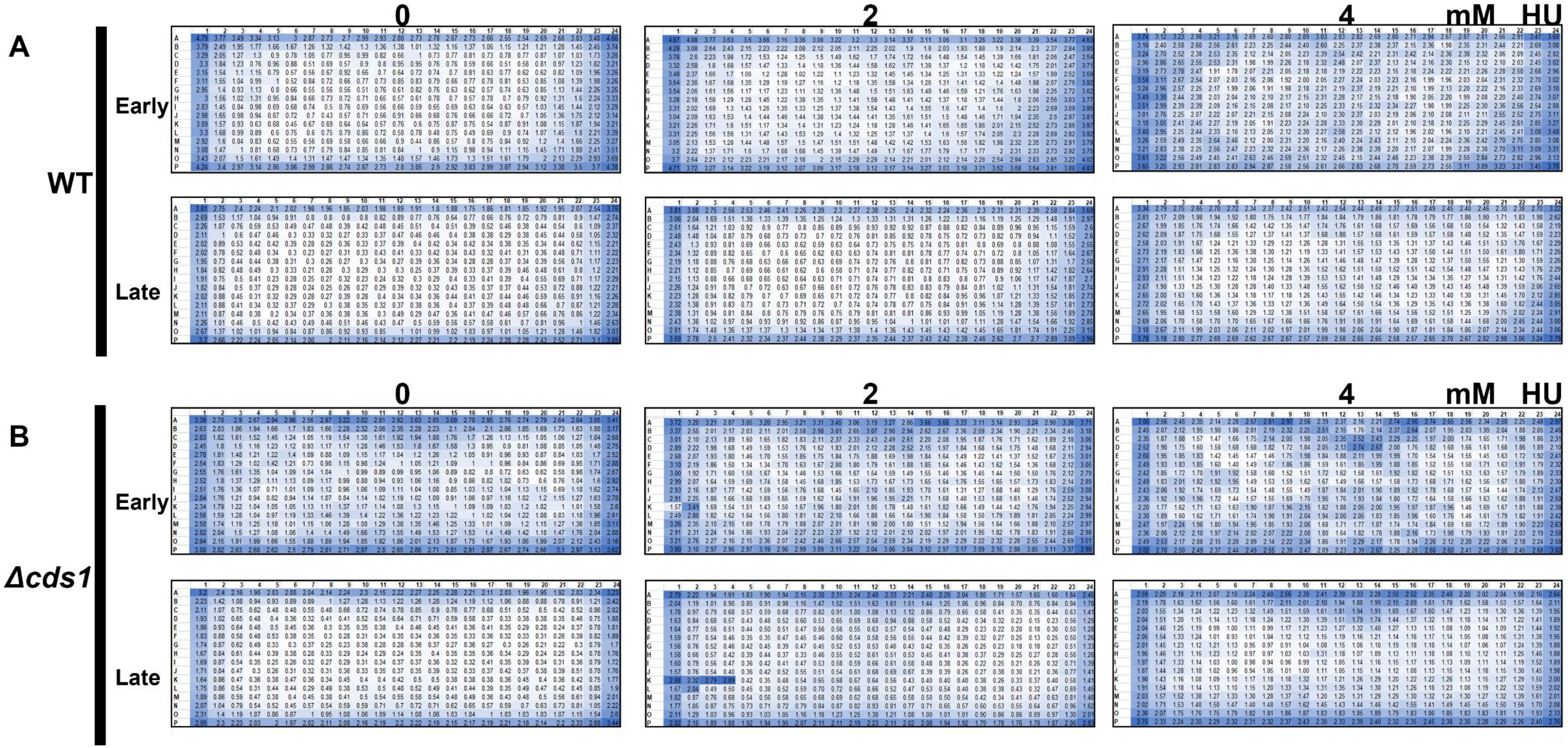
0
2
4 mM HU
A
WT
Early
Late
0
2
4 mM HU
B
Δcds1
Early
Late

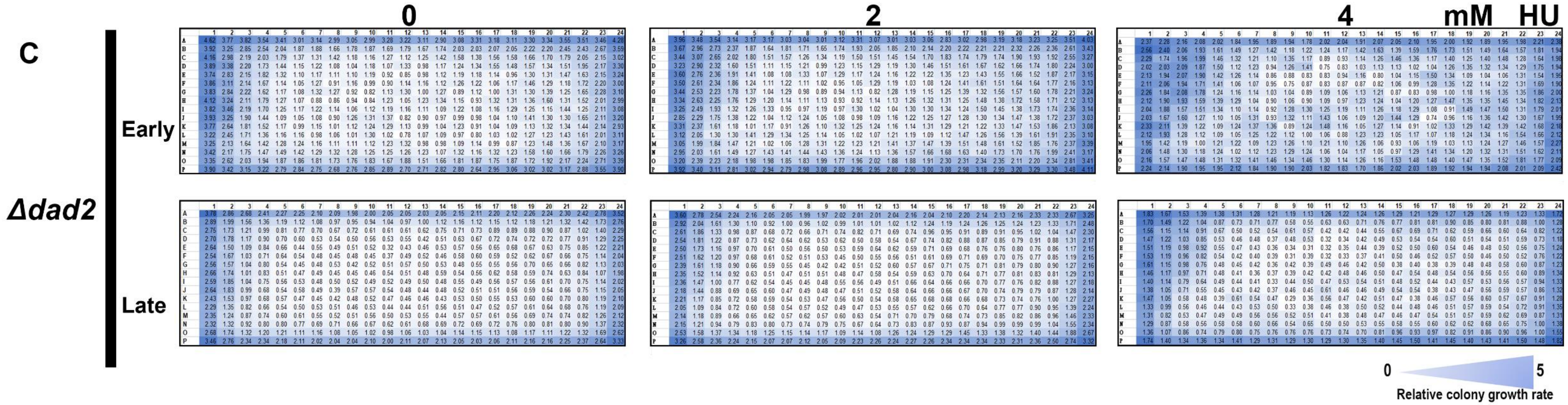

Figure S8. Extensive edge effect was detected with heatmaps of growth rate at 384-spot setting at both growth phase and all HU concentrations. (A) WT, (B) *Δcds1* and (C) *Δdad2* cells were pinned onto agar plate at spot density of 384 with 0, 2 and 4mM HU. Early and late growth rate were calculated from the slope of growth curve generated from the PhenoSuite® and organized into heatmaps with annotation of relative growth rate at different loci. Prominent edge effects were observed at plates pinned at density of 384 in term of colony growth rate, regardless of drug concentration or yeast strains used.

biomedicines

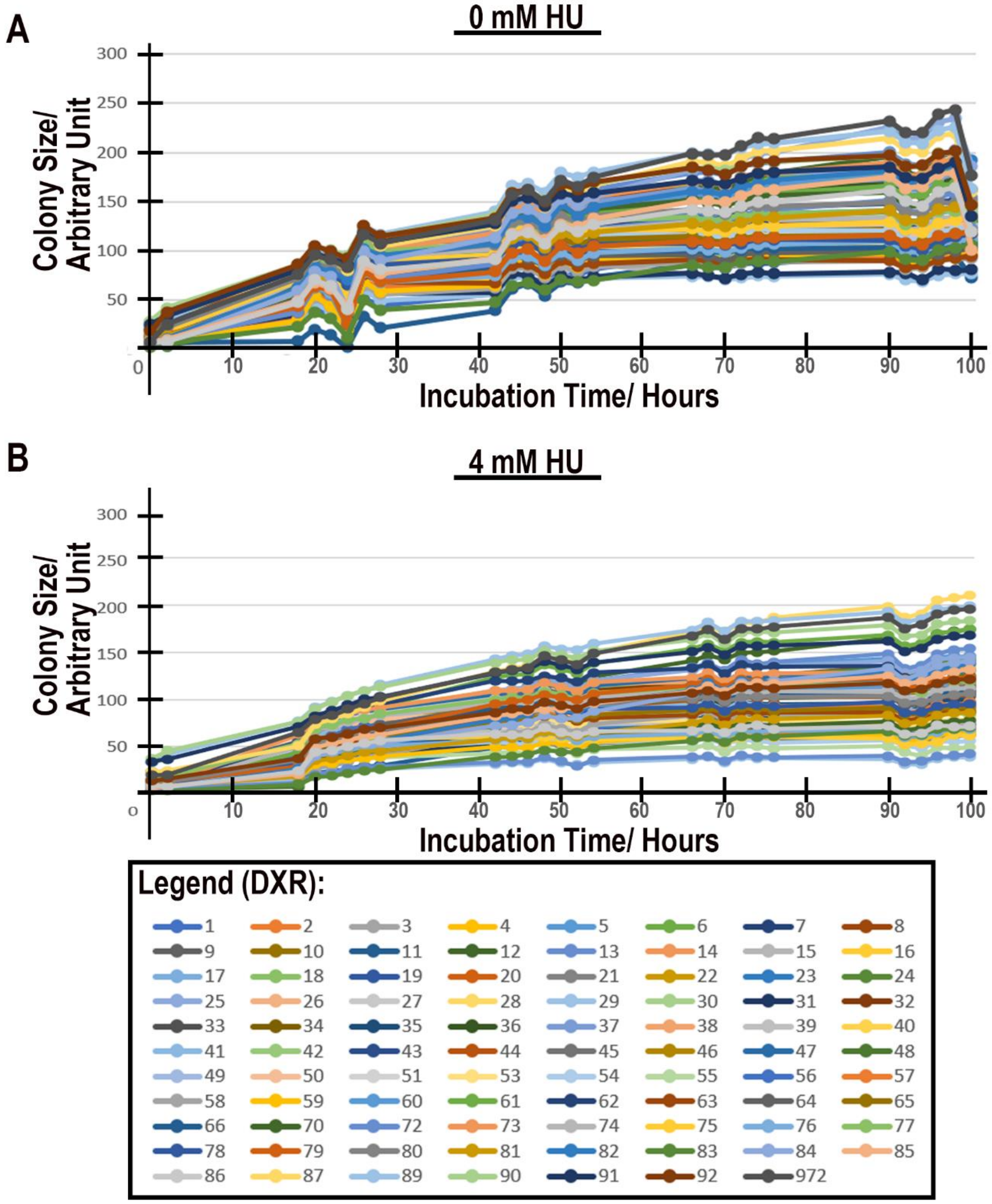

Figure S9. ROTOR-based HU screening at 384-spot setting. (A) Example of growth curves of all MER strains pinned on control plate (without HU) at 384-spot setting. (B) Example of growth curves of all MER strains pinned on 4mM HU plate at 384-spot setting.

A

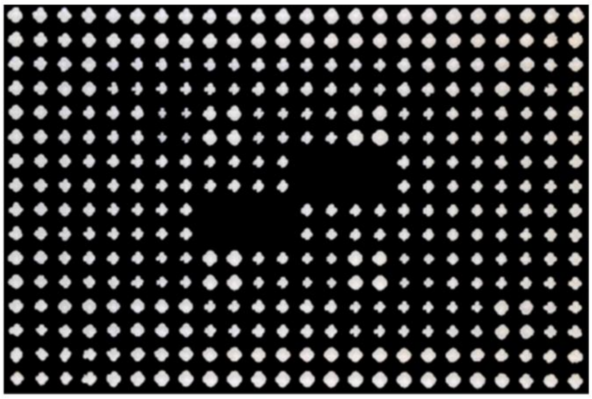

B

	1	2	3	4	5	6	7	8	9	10	11	12	13	14	15	16	17	18	19	20	21	22	23	24
A	3.96	3.47	3.16	3.30	3.27	3.35	3.18	3.44	3.31	3.26	3.32	3.22	3.43	3.09	3.33	3.21	3.03	3.09	2.83	2.87	2.83	2.97	3.18	3.23
B	3.39	2.34	2.25	2.31	2.23	2.10	2.48	2.76	2.52	2.67	2.29	2.61	2.55	2.37	2.35	2.20	2.30	1.98	1.71	1.84	1.77	1.65	2.37	3.67
C	2.91	1.85	1.65	1.67	1.58	1.95	2.15	2.22	2.52	2.32	2.30	1.84	2.07	2.01	1.87	1.81	1.54	1.24	1.18	1.10	1.18	1.32	1.69	2.83
D	2.70	1.77	1.07	1.19	1.83	2.40	2.48	2.42	2.48	2.33	2.34	2.48	2.43	2.27	2.07	2.09	1.55	1.93	1.49	1.35	1.36	1.21	1.76	2.79
E	2.72	1.56	1.31	1.47	1.61	2.14	2.81	2.90	2.35	2.51	3.03	3.67	3.17	2.90	2.28	2.03	2.26	2.27	1.83	1.68	1.76	1.39	1.58	2.86
F	2.72	1.39	1.29	1.37	1.79	2.13	2.85	3.36	2.26	2.29	4.07	3.98	3.68	3.56	2.92	2.55	2.44	2.64	2.16	2.09	2.06	1.56	2.29	2.73
G	2.53	1.61	1.44	1.10	1.83	1.96	2.28	2.89	2.69	2.77	3.18	3.49	0.00	0.00	0.00	0.00	2.88	2.86	2.67	2.05	2.17	1.81	2.10	2.86
H	2.66	1.48	1.30	1.35	1.67	1.96	2.16	2.54	3.10	3.21	3.77	3.91	0.00	0.00	0.00	0.00	3.05	2.66	2.55	2.22	2.28	1.69	2.09	2.58
I	2.69	1.62	1.37	1.57	1.57	1.85	2.16	2.67	0.00	0.00	0.00	0.00	3.94	3.85	3.44	3.33	3.20	2.69	2.38	2.22	2.03	2.13	2.09	2.49
J	2.70	1.65	1.36	1.40	1.68	1.81	1.97	2.50	0.00	0.00	0.00	3.40	3.37	3.13	3.11	3.01	2.81	2.63	2.41	2.41	2.10	2.03	2.05	2.71
K	2.75	1.73	1.28	1.48	1.72	1.66	1.85	2.04	2.62	2.66	3.17	3.24	3.37	3.26	2.33	2.50	2.79	2.87	2.58	2.27	2.08	1.95	2.13	2.71
L	2.92	1.84	1.29	1.22	1.28	1.47	1.77	1.97	1.63	2.05	2.63	3.02	3.02	2.89	2.48	2.22	2.88	2.99	2.27	2.31	1.98	1.90	1.88	2.75
M	2.71	1.70	1.30	1.32	1.26	1.27	1.26	1.61	1.74	1.95	2.06	2.27	2.20	2.23	2.42	2.35	2.47	2.18	2.38	2.27	1.90	1.85	2.12	2.62
N	2.62	1.64	1.54	1.38	1.33	1.39	1.49	1.36	1.57	1.81	1.85	1.78	2.04	1.93	2.32	2.42	2.42	2.45	2.31	1.89	1.74	1.81	1.85	2.98
O	3.21	2.32	1.99	2.05	2.25	2.07	2.08	2.33	1.96	2.33	2.37	2.38	2.53	2.54	2.69	2.56	2.41	2.54	2.58	2.39	2.06	2.29	2.52	3.37
P	3.23	2.93	2.46	3.25	3.18	3.27	3.24	3.00	3.41	3.43	3.52	3.55	3.61	3.74	3.56	3.57	3.65	3.47	3.51	3.64	3.21	3.26	3.28	4.00

0 5
Relative colony growth rate

Figure S10. Skipping pinning at the center of agar plates created an additional 'edge'. (A) A snapshot illustrating position of two "inner edges", and growth pattern after 57 h of incubation at 30°C**.** WT cells were pinned at 384-spot setting on a control plate, except two defined loci located at the center of the agar plate. (B) A heatmap of relative growth rate of WT cells indicating impacts of edge effects towards colonies located around the two defined loci without cell pinning (four-fold higher relative growth rate as compared to other WT cells in neighboring loci.

A

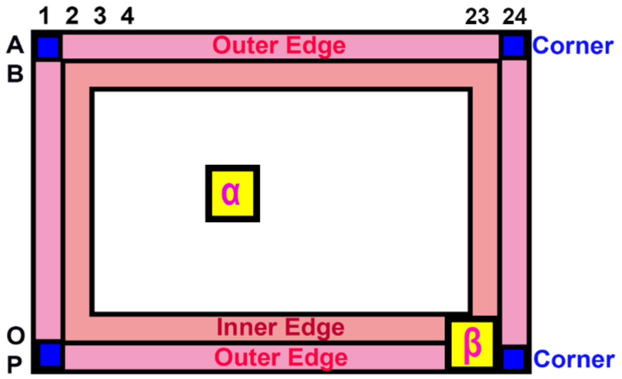

B

WT	Early Growth Rate	Late Growth Rate
α	0.17	0.09
β	0.22	0.29

Figure S11. Control strains pinned at center and outer edge show differences in relative growth rate. (A) A map illustrating the relative position of two clusters of WT cells (α and β) pinned together with other MER strains in a 384-spot setting on a control plate. (B) The two clusters of WT cells gave approximately 2 folds differences in relative growth rate, which may give a misleading result in drug sensitivity determination based on comparison with values from WT controls.
